# Stem Cell Studies in Cardiovascular Biology and Medicine: A Possible Key Role of Macrophages

**DOI:** 10.3390/biology11010122

**Published:** 2022-01-12

**Authors:** Nanako Kawaguchi, Toshio Nakanishi

**Affiliations:** Department of Pediatric Cardiology and Adult Congenital Cardiology, Tokyo Women’s Medical University, Tokyo 162-8666, Japan; nakanishi.toshio@twmu.ac.jp

**Keywords:** induced pluripotent stem cell, iPSC, bone marrow stem cell, adipose-derived stem cell, exosome, macrophage, chemokine, CXCR4, inflammation

## Abstract

**Simple Summary:**

Stem cells are used in cardiovascular biology and biomedicine and this field of research is expanding. Two types of stem cells have been used in research: induced pluripotent and somatic stem cells. Induced pluripotent stem cells (iPSCs) are similar to embryonic stem cells (ESCs) in that they can differentiate into somatic cells. Bone marrow stem/stromal cells (BMSCs), adipose-derived stem cells (ASCs), and cardiac stem cells (CSCs) are somatic stem cells that have been used for cardiac regeneration. Recent studies have indicated that exosomes and vesicles from BMSCs and ASCs can be used in regenerative medicine and diagnostics. Chemokines and exosomes can contribute to the communication between inflammatory cells and stem cells to differentiate stem cells into the cell types required for tissue regeneration or repair. In this review, we address these issues based on our research and previous publications.

**Abstract:**

Stem cells are used in cardiovascular biology and biomedicine, and research in this field is expanding. Two types of stem cells have been used in research: induced pluripotent and somatic stem cells. Stem cell research in cardiovascular medicine has developed rapidly following the discovery of different types of stem cells. Induced pluripotent stem cells (iPSCs) possess potent differentiation ability, unlike somatic stem cells, and have been postulated for a long time. However, differentiating into adult-type mature and functional cardiac myocytes (CMs) remains difficult. Bone marrow stem/stromal cells (BMSCs), adipose-derived stem cells (ASCs), and cardiac stem cells (CSCs) are somatic stem cells used for cardiac regeneration. Among somatic stem cells, bone marrow stem/stromal cells (BMSCs) were the first to be discovered and are relatively well-characterized. BMSCs were once thought to have differentiation ability in infarcted areas of the heart, but it has been identified that paracrine cytokines and micro-RNAs derived from BMSCs contributed to that effect. Moreover, vesicles and exosomes from these cells have similar effects and are effective in cardiac repair. The molecular signature of exosomes can also be used for diagnostics because exosomes have the characteristics of their origin cells. Cardiac stem cells (CSCs) differentiate into cardiomyocytes, smooth muscle cells, and endothelial cells, and supply cardiomyocytes during myocardial infarction by differentiating into newly formed cardiomyocytes. Stem cell niches and inflammatory cells play important roles in stem cell regulation and the recovery of damaged tissues. In particular, chemokines can contribute to the communication between inflammatory cells and stem cells. In this review, we present the current status of this exciting and promising research field.

## 1. Introduction

The incidence of myocardial infarction has increased worldwide; however, heart transplantation is the only fundamental solution. Therefore, stem cells, which are easy to handle and differentiate, have been investigated. Two decades ago, adult bone marrow stem cells (BMSCs) were reported to have the ability to develop into cardiac myocytes [1]. Multiple studies have replicated this finding, and BMSCs have been transplanted into infarcted hearts to generate differentiated cardiomyocytes. These studies have laid the foundation for discussions on whether these cells have an efficient capacity to differentiate into functional cardiac myocytes, and how to use them efficiently in clinical settings. In 2003, the existence of c-kit-positive cardiac stem cells (CSCs) in the adult rat heart was reported [2], catalyzing the interest in further study of these fascinating cells. Other cardiac stem cell markers, such as stem cell antigen-1 (sca-1), ATP-binding cassette subfamily G, member 2 (abcg2), and islet-1, have also been explored [3]. The formation of the cardiosphere is considered a characteristic of CSCs. However, the efficiency of differentiation of most adult somatic stem cells into cardiac myocytes is low. In 2006, induced pluripotent stem cells (iPSCs), which are similar to embryonic stem cells (ESCs), were established using murine [4] and human [5,6] fibroblasts. Due to their potent ability to differentiate into functional, beating cardiac myocytes, a property that has not been observed in somatic stem cells, iPSCs have been widely used, not only in regenerative medicine but also in in vitro disease models to evaluate the druggability of different chemicals for preclinical studies. Previous studies have characterized somatic stem cells [7] and iPSCs [4] and compared their similarities and differentiation capabilities in the field of cardiovascular research [8]. Indeed, Pushp et al. demonstrated that functional beating cardiac myocytes were formed using iPSCs but not from umbilical cord-derived mesenchymal stromal stem cells. In addition, the regulation of the stem cell environment, particularly stem cell niches and chemokines, has also been investigated [9,10]. Chemokines are associated with inflammation and cell-cell interactions between niches and stem cells. Recently, inflammatory cells have received attention not only for immunological responses but also for triggering tissue healing [11,12,13,14]. Here, we review recent studies in the cardiovascular field focusing on these stem cells, chemokines, and inflammatory cells, and discuss current achievements and areas of future development. Furthermore, we focus on macrophages, which have been reported to play an important role in recent studies. In this review, we have addressed stem cell studies in cardiovascular biology and medicine based on our experiments and other previously reported studies.

## 2. Induced Pluripotent Stem Cells (iPSCs)

Ideally, stem cells used in cardiovascular regenerative medicine should be able to easily differentiate into functional cardiomyocytes. The iPSCs and ESCs are the most suitable in that sense, although iPSCs are more ethically and immunologically less concerning because of their lower immuno-rejection. The generation of iPSCs holds great promise for cardiac regenerative medicine because iPSCs resemble ESCs, which are known to differentiate into spontaneously beating cardiac myocytes and other types of cardiac cells, such as endothelial cells, smooth muscle cells, and cardiac fibroblasts [15]. The iPSCs can be generated from any cell type with the potential to proliferate. For example, skin dermal fibroblasts were originally used, but noninvasive cells were preferred. Therefore, peripheral blood cells [16,17,18,19,20,21,22], including T-cells [22] and B-cells [20], have been used to generate iPSCs. Epithelial cells isolated from urine have been used as a less invasive method of sample collection to generate iPSCs [23]. These cells are not invasive and show cardiac differentiation similar to that of other cells [24,25,26,27].

Pluripotency is also associated with cancer; ESCs are known to cause cancer in vivo. The use of the *c-myc* oncogene increased the efficiency of inducing iPSCs; however, it may increase the cause of cancer when transplanted into the body. Therefore, efforts have been made to generate iPSCs without introducing *c-Myc/KLF4*, for example, by using microRNAs [28]. The use of retroviral vectors has been changed to Sendai virus (without integration into the host genome) [29] or no vectors have been used for safety [30]. The xeno-free culture condition was developed for safer iPSC generation [31]. Extracellular vesicles from iPSCs were found to be efficient for cardiac repair [32]. Thus, efforts have been made for the safe usage of iPSC culture for future regenerative medicine.

To efficiently transplant iPSC-derived cardiomyocytes (iPSC-CMs) into damaged tissues, three-dimensional (3D) structures containing iPSC-CMs in cell sheets or scaffolds have been developed [33,34,35]. These cells and the 3D structures carrying them are expected to be used not only for regenerative medicine but also in in vitro disease models since only animal disease models are currently available for studying drug efficacy and toxicity. Recently, we established a rat model of pulmonary arterial hypertension (PAH) disease by monocrotaline injection (to cause inflammation) and maintained hypoxia in rats, which may serve as a good experimental animal model. We studied the effects of silibinin [36,37,38], an inhibitor of chemokine receptor, CXC motif chemokine receptor type 4 (CXCR4), and found that CXCR4 expression was higher in PAH rats [37]. The pulmonary artery becomes thick, causing hypertrophy of the right ventricle, leading to death in the worst cases [39]. We found that silibinin significantly reduced right ventricular pressure. However, we still do not know the mechanism of action and do not know whether silibinin is effective in humans. Thus, disease models based on human cells are required. The iPSCs have also been used to establish disease models. However, it is difficult to develop an in vitro model for PAH because it requires human iPSC-CMs (hiPSC-CMs), lungs, pulmonary arteries, and their connections. PAH affects multiple organs and tissues and one of the barriers to establishing this multi-complex is the requirement of a circulatory system that supplies oxygen and nutrients to each component and removes the waste. This type of system is necessary to maintain cell viability, and for this purpose, technologies using cell sheets [40], collagen scaffolds including decellarized matrix [41], and other synthetic polymers have progressed [42]. Thus, useful models may be established using these technologies and have recently revived ideas of self-organizing embryonic and cardiac organoids mimicking the physiological developmental process of heart generation in the future [43].

Current in vitro disease models target diseases that arise from malignancies of single-cell types or point mutations, including long QT syndrome caused by a mutation in the *SCN5A* gene, which encodes the sodium channel and other channels such as potassium channels (including *KCNJ2, KCNJ5,* and *KCNE1*) [44]. There are other in vitro disease models, but they have been reviewed by us previously [33] and Kamga et al. more recently [45]. LQTS may be suitable for establishing a model for diseases caused by mutations in ion channels that are directly associated with cardiomyocyte beating. Indeed, an in vitro disease model was established to analyze this dysfunction [46,47] soon after functional cardiac myocytes were successfully differentiated from patient iPSCs [48,49]. However, differences between cell lines have been reported [50], and temporal changes in hiPSC-CM phenotypes have also been reported [51]. Additionally, variations in the results were observed even when using the same cell source, irrespective of whether the cells were commercially procured [52,53]. Moreover, hiPSC-CMs exhibited different characteristics in their aggregates and single states. Shah et al. used an in silico model to show that aggregates more closely represent the clinical phenotype than single cells. They recommended the use of a 3D-culture system to establish an in vitro disease model, as it more closely resembles in vivo models [54]. A 3D model can be more natural than a conventional 2D culture. Cardiac microtissues (MTs) and engineered cardiac tissues have been developed. Non-cardiac myocytes, such as cardiac fibroblasts and endothelial cells, were found to be more important for the development of mature hiPSC-CMs than hiPSC-CMs alone [15]. Mature hiPSC-CMs are more developed using various non-cardiac myocyte cells than using only one of the non-cardiac myocyte cells [15]. Furthermore, the surrounding extracellular matrix (ECM) contributes to cardiomyocyte maturation [55]. Ozcebe et al. showed that aged ECM impairs cardiac function, whereas adult ECM promoted cardiac function [56]. Taken together, these studies show that in vitro disease models that mimic in vivo systems and are sufficiently complex to deal with multiple tissues or organs will need to be developed in the future.

The hiPSC-CMs are more immature than the adult hCMs. Cardiomyocytes appear early in the embryonic stage and it takes time for them to mature into adult cardiomyocytes. Thus, immature cardiomyocytes mature in long-term culture [51]. Various methodologies have been developed for generating mature cardiac myocytes or selecting mature cardiomyocytes. For example, Tohyama et al. exploited the metabolic differences between iPSCs and iPSC-CMs, which can use lactate in the absence of glucose [57]. Dubois et al. identified a specific cell surface marker, SIRPA, for isolating differentiated iPSC-CMs [58]. For safe use of iPSC-CMs in regenerative medicine, the separation of fully developed cardiomyocytes is important for the removal of undifferentiated cells that may cause cancer.

Another approach to developing mature hiPSC-CMs involves the development of a 3D system containing iPSC-CMs and other cell types of the same origin. Lange et al. constructed engineered cardiac tissues that form t-tubules, which are more similar to human tissues than 2D cultures [59]. Additionally, Masumoto et al. reported that engineered cardiac tissues ameliorated myocardial dysfunction [60]. Several symptoms of hypertrophic cardiomyopathy and Noonan syndrome cause hypertrophy of cardiomyocytes and have been investigated and analyzed using the CRISPR/Cas system to make corrections or generate mutated genes [61]. Hanses et al. generated clear data showing that its pathology is correlated with the RAS mitogen, which is activated by hiPSC-CM. Overall, changes in cell structure or shape, such as hypertrophy, could be a more successful target for in vitro disease models [61]. Mitochondrial hypertrophy disease models established from patient iPSC-CMs have also been recently reviewed [62].

**Figure 1:**
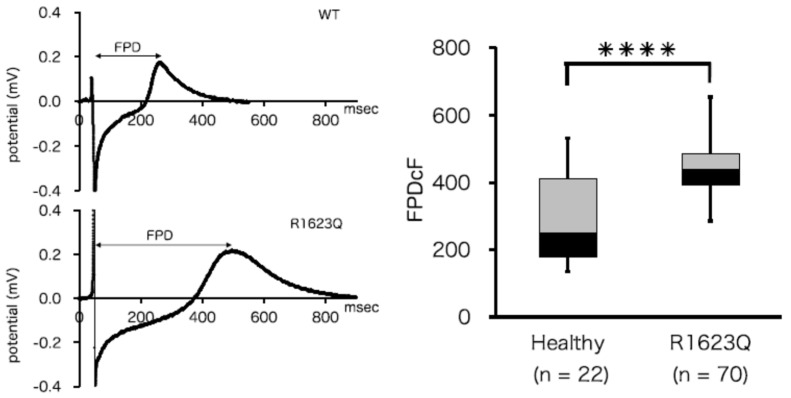
Electrophysiological analysis of induced pluripotent stem cells (iPSCs)-derived cardiomyocytes (CMs). (Left panel) Representative baseline field potential waveforms in iPSC-CMs from healthy volunteers (upper) and LQT syndrome type 3 (LQT3) patients with the R1623Q *SCN5A* mutation (lower). (Right panel) The field potential duration corrected by Fridericia’s correction formula (FPDcF) of R1623Q mutation-harboring hiPSC-CMs was significantly larger than that of WT iPSC-CMs (from [49]).**** *p* < 0.0001.

Our laboratory previously developed hiPSC-CMs from patients with LQT syndrome type 3 (LQT3) and healthy volunteers [63]. LQT-3 is caused by a mutation in sodium channel *SCN5A,* which increases the inward sodium current [44]. LQT-3 patient (R1623Q)-derived hiPSC-CMs had a larger field potential duration than healthy controls, which showed a similar phenotype to the patient (Figure 1). We investigated how the neonatal splicing form of *SCN5A* contributes to LQT3 disease and found that it affects the severity of the disease. However, it was difficult to analyze iPSC-CMs electrophysiologically, which may have been due to the heterogeneity of the patient chromosomes. Therefore, we used *SCN5A* transfected cell lines. Subtle changes are difficult to determine in the current heterogeneous hiPS-CM situation. Thus, there is still a limitation in using iPSC-CMs as an in vitro disease model.

A comprehensive in vitro proarrhythmia assay (CiPA) was established by researchers at national research institutes in collaboration with pharmaceutical companies in several countries to evaluate the arrhythmia effect of drugs to determine whether it causes torsades de pointes (TdPs), as not all drugs that cause longer QT cause TdPs [64,65,66,67]. The hiPSC-CM is potentially a good tool for evaluating this effect [68]. Yim summarized this project [69]. We previously identified variability in the characteristics of harvested c-kit-positive CSCs derived from rat hearts, even when the same protocol was used [70]. Thus, we must consider the heterogeneity between stem cells, in addition to possible technical errors and individual differences.

## 3. Somatic Stem Cells

### 3.1. Bone Marrow Stem Cells (BMSCs) and Mesenchymal Stem Cells (MSCs)

BMSCs were the first somatic stem cells to be identified as multipotent, with the ability to differentiate into mesenchymal cells such as adipocytes and osteoblasts [71]. Soon after this discovery, BMSCs were also reported to have the ability to differentiate into cardiomyocytes in vivo and in vitro and alleviate myocardial infarction [1]. Since then, these cells have been extensively studied for their clinical and preclinical applications. BMSCs also contain stem/progenitor cells of hematopoietic and mesenchymal stem cells that are associated with angiogenesis-producing paracrine factors. Recent research has focused on paracrine factors and vesicles released from these cells. MSCs are present in the bone marrow (BM) and various other organs. Endothelial progenitor cells release various cytokines and growth factors, including vascular endothelial growth factor (VEGF), which is found in the peripheral blood [72]. However, these cells do not originate from the BM but from resident niches identified following sex-mismatched transplantation [73].

The positive effect of BMSC transplantation on myocardial infarction is attributed to its paracrine effects. Efforts have been made to improve the ability of BMSCs to treat myocardial infarction through (1) isolation of specific cell types such as CD133 [74], CD271 [75], and CD117 (c-kit)-positive cells; (2) genetically engineered cells overexpressing VEGF [76], hepatocyte growth factor (HGF) [77], and insulin-like growth factor (IGF) [78]; (3) use of exosomes derived from BMCs; (4) using microRNAs such as miR-19a/19b [79] and miR-29a [80]; and (5) using 3D structures of BMCs or engineered BMCs and exosomes.

Extracellular vesicles (EVs) and exosomes (exos) from BMSCs have been well-characterized. MSC-EVs and MSC-exos are approximately 100 nm in diameter, contain micro-RNA and mRNA, and express CD9, CD63, and CD81, which act as surface markers of their extracellular domains. They have been shown to have a curative effect on myocardial infarction [81]. MSC-exos also attenuate cardiac hypertrophy and fibrosis [82]. Fu et al. showed that miR-338 in MSC-exos cured myocardial infarction by inhibiting cardiomyocyte apoptosis [83]. Moreover, MSCs from the umbilical cord (UC) have more positive effects [84]. Zhang et al. also found that UC-MSC-exos could rejuvenate aged BM-MSCs, most likely via miR-136, by targeting apoptotic protease activating factor-1 (Apaf1) [84]. MSC-exos are eventually internalized by neighboring cells. Overall, UC-derived MSCs were more potent than BM-derived MSCs. The efficacy of MSCs derived from adipose tissue has also been studied and is discussed in the next section.

The fate of stem cells depends on the ECM, which determines substrate stiffness [85,86]. Computation is a useful tool for regulating this parameter. Urdeitx and Doneider used a piezoelectric fibered extracellular matrix in a 3D computational model [87] to calculate the intracellular force affected by the fiber. Computational models can be powerful tools for estimating precise cellular changes that connect differentiation.

### 3.2. Adipose-Derived MSCs

Similar characteristics have been identified in MSCs derived from adipose tissue and BM. For example, both cells have the capacity to differentiate into multiple cell types. They also release growth factors and cytokines, although not the same factors [88]. MSCs circulate in or exist in tissue niches surrounded by low oxygen levels. Cell populations and characteristics also differ between adult and neonatal tissues [89]. Adolfsson et al. compared MSCs derived from the BM with those derived from adipose tissue. They found that adipose-derived MSCs proliferated more than BM-derived MSCs, and adipose-derived MSCs had higher *angiopoetin 1* (*angpt1)*, *Leukemia inhibitory factor* (*LIF)*, and *Transforming growth factor* (*TGF)-β1* expression levels, but equal *VEGF-A* and *HGF* expression levels compared with BMSCs [90]. Conditioned medium from adipose-derived stromal cells has also been studied because of its positive ameliorating effect on various damaged tissues, including the infarcted heart, which occurs through paracrine factors [91]. Lu et al. fractionated conditioned media based on molecular weight and found that fractions over 50 kD protected the endothelium from barrier dysfunction caused by H_2_O_2_ and fractions less than 3 kD protected against apoptosis induced by tumor necrosis factor (TNF)-α [92]. Lai et al. reported that exosomes from conditioned media from adipose-derived MSCs contained high levels of miR-221/222 and attenuated myocardial infarction in a mouse model. Knockout of miR-221/222 in mice increased apoptosis and fibrosis; however, treatment with conditioned medium from adipose-derived MSCs decreased apoptosis and fibrosis [93]. Lee et al. found that intramuscular injection of conditioned media from adipose-derived MSCs attenuates ischemia in mice [94]. Taken together, exosomes from adipose-derived stromal cells ameliorated ischemia through the action of miR-221/222.

Attempts have been made to differentiate adipose-derived stem and stromal cells into cardiomyocytes. Seheli et al. reported that 5-azacytidine, a DNA methyltransferase inhibitor, played a role in this differentiation [95]. Darche et al. reported that adipose-derived stem/stromal cells can function as pacemaker cells [96]. However, Stepniewski et al. compared the abilities of iPSC-CMs and adipose-derived stem/stromal cells (derived CMs) to cure myocardial infarction and demonstrated better outcomes with iPSC-CMs [97].

### 3.3. Cardiac Stem Cells (CSCs)/Cardiac Progenitor Cells (CPCs)

Cardiac stem cells (CSCs) were originally found to be lineage (-) c-kit-positive cells in adult rat hearts [2]. CSCs have been reported to differentiate into small cardiomyocytes when cultured in a differentiation medium. Other cell markers, such as Sca-1 and abcg2, have also been found in adult rat hearts. The formation of the cardiosphere was also found to be a characteristic of CSCs, and clinical studies for post-myocardial infarction treatment using cardiosphere-derived cells have been performed and improvement was observed [98,99]. Cardiosphere-derived cells were found to grow as self-adherent clusters from subcultures of postnatal atrial or ventricular human biopsy specimens and from murine hearts [100,101]. These cardiac stem cell studies were reviewed by Matsa et al. [3]. Islet-1 has been found to be a distinct cardiac lineage cell progenitor in embryonic and neonatal mice and human hearts [102,103]. Interestingly, a recent study suggested that Islet-1 leads Gcn5 to bind to the *GATA4/Nkx2.5* promoter region, which promotes cardiomyocyte differentiation in BMSCs [104]. Therefore, the introduction of *Islet-1* into somatic stem cells has the potential to produce cardiomyocytes.

The existence of CSCs, which have recently been termed cardiac progenitor cells (CPCs), has been discussed since their discovery. As previously confirmed by us, c-Kit positive cells exist in adult rat hearts [105], and we isolated and characterized them after long-term culture and observed cardiac progenitor and BMSC characteristics [71]. Ellison et al. reported a modified method for the isolation of CSCs and showed that sufficient amounts of endogenous CSCs can be isolated from rats with heart injury using high-dose isoproterenol [106]. They also found that c-kit was not sufficient to enhance myogenesis, but other selection markers could [107]. Although these findings facilitate this area of research, discussions are ongoing. Recently, Vegnozzi et al. suggested that the positive repair effect of cardiac stem cell implantation for repairing damaged tissues could be induced by resident macrophages [11], as described in the next section. Hoving et al. isolated human CSCs (hCSCs) from the cultured tissue debris in a CSC medium and characterized their migration behavior in human serum. They found that CSC migration caused by human serum was inhibited by a p-38 MAPK inhibitor [108]. We isolated these cells in a similar manner, but by using a c-kit antibody after collecting the cells which were migrated from the tissue debris, and obtained multipotent stem cells [109], which were used for differentiation analysis [110]. Thus, the isolation methodology can be continuously modified and improved.

## 4. Stem Cell Microenvironment and Macrophage Involvement

The microenvironment that maintains stem cell quiescence in the BM is facilitated by niches consisting of CXC chemokine ligand (CXCL)12-abundant reticular (CAR) cells. CAR cells express high levels of CXCL12/SDF-1, stem cell factor (SCF), forkhead box C1 (FOXC1), and early B cell factor 3 (EBF3) in the murine BM. CAR cells have been identified in humans, and patients with chronic myeloid leukemia have reduced levels of these factors [111]. Chemokines such as CXCR4 play important roles in the migration and maintenance of these niches [112]. We previously observed the proliferation of CXCR4+ inflammatory cells in cultured BMCs using silibinin (CXCR4 antagonists), particularly when inflammation was activated [38]. Chemokines can function either positively or negatively during wound healing and tissue repair [9]. Interestingly, silibinin increases macrophage and neutrophil counts in cultured BM cells [38]. We first hypothesized that silibinin ameliorated PAH because it may bind CXCR4 positive inflammatory cells and inhibit these cells. However, since there are anti-inflammatory resident macrophages as we describe below, we now consider that silibinin may affect resident macrophages during damage healing.

Accumulating evidence has suggested that macrophages play an important role in stem cell regulation. In skeletal muscles, pax3 expressing muscle stem cells (MuSCs) differentiate into muscle cells following injury. Macrophages transiently migrate to the wound site, and dwelling macrophages are associated with MuSCs. Ablation of dwelling macrophages leads to a reduction in MuSCs [113]. Dwelling macrophages secrete nicotinamide phosphoribosyltransferase (Nampt), which stimulates myoblast proliferation. Interestingly, the C-C motif chemokine receptor 5 (Ccr5), a receptor for Nampt, is expressed by MuSCs. Thus, Nampt is hypothesized to function in muscle regeneration and is a potential therapeutic target. Furthermore, Vagnozzi et al. suggested that macrophages are key regulators in the healing of damage caused by an infarcted myocardium [11]. Attenuation of the infarcted heart was limited to the absence of CCR2+ and CX3CR1+ macrophages. Tissue-specific macrophages have also been identified [114]. Macrophages in the heart are heterogeneous and contain CCR2+ and CX3CR1+ subpopulations [115]. Different macrophage subpopulations can express different cell surface proteins and may have different functions [115], as is the case in the human system [116]. They can act either positively or negatively during the healing of damaged tissues.

### Macrophages in the Heart

Resident cardiac macrophages that originate from the yolk sac or fetal liver during embryonic development are characterized by Ccr2− and MHC II lo/hi, whereas those that originate from the bone marrow during postnatal development are characterized by Ccr2+ and MHC II lhi [117,118]. Resident macrophages exert anti-inflammatory and antifibrotic effects in injured hearts. Inflammation causes fibrosis in the heart, resulting in arrhythmia. Bajpai et al. showed that tissue-resident Ccr2− macrophage-deficient mice had larger infarct sizes than control mice, while tissue-resident Ccr2+ macrophages could cause inflammation by promoting monocyte recruitment [119]. Monocytes can develop into macrophages, particularly during inflammation. Interestingly, they postulated that the population of tissue-resident Ccr2+ macrophages increases with age, causing further inflammation in the heart. Exosomes can also contribute to recovery from myocardial infarction and inhibit fibrosis [120]. Myocardial infarction biomarkers include specific miRNAs for the early diagnosis of hypertrophic cardiomyopathy (miR-21, miR425, and miR-744) and heart failure (miR34a, miR192, and miR-194), which are released by exosomes [120]. Intracellular communication has also recently been considered as a factor [121]. Extracellular vesicles from cardiac-derived adherent proliferating (CardAP) cells enhance angiogenesis in human umbilical vein endothelial cells (HUVECs) [122]. Angiogenesis can also play a role in recovery from myocardial infarction through the supplementation of oxygen and nutrients to the infarcted area.

**Figure 2:**
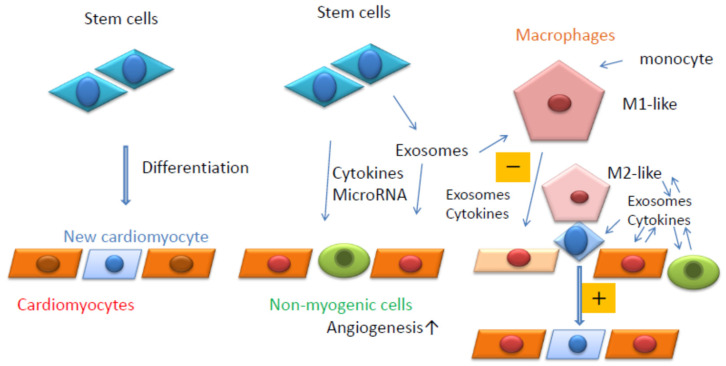
Summary of the current review. Stem cells can differentiate into cardiomyocytes (**Left**). Stem cells can release cytokines, microRNAs, and exosomes. Exosomes also contain cytokines and microRNAs (**Middle**). Resident macrophages can contact stem cells in close proximity to cardiomyocytes and induce their differentiation into cardiomyocytes (**Right**). Resident macrophages and monocyte-derived macrophages are affected by exosomes secreted by surrounding cells and can affect the surrounding cells positively (M2-like, +) or negatively (M1-like, −) (**Right**).

Macrophages are classified as M1 or M2; M1 macrophages are inflammatory, whereas M2 macrophages are anti-inflammatory in nature. Exosomes from ESCs reduce the inflammation caused by doxorubicin (DOX)-induced cardiotoxicity, which can lead to heart failure [123] and an increase in M2 macrophages. DOX is an effective antineoplastic agent with adverse cardiotoxic effects. Macrophages secrete exosomes containing miR-155, which promote inflammation during cardiac injury [124]. Wang et al. found that miR-155 in cardiac fibroblasts was derived from exosomes secreted by macrophages [124]. In contrast, exosomes derived from M1-like macrophages are often secreted after myocardial infarction and promote cardiac dysfunction. Regenerative medicines that inhibit M1-like macrophages or enhance M2-like macrophages can be developed as potential treatments. Taken together, macrophages can function as key regulators, receiving signals from exosomes or cytokines secreted by myogenic or non-myogenic cells (Figure 2). Therefore, macrophages are likely to be receiving increased attention in regenerative medicine.

## 5. Conclusions

Cardiomyocytes differentiated from iPSCs are immature. Efforts have been made to obtain mature cardiomyocytes from iPSCs by using cell surface markers and/or metabolic differences between iPSCs and cardiomyocytes. In vitro disease models with more complex structures can be developed using a circulating system to nurse the cells. Somatic stem cells are inferior to iPSCs in terms of their differentiation capability; however, recent studies have shown that exosomes and microvesicles may be used for cardiomyocyte regeneration. Exosomes contain microRNAs and cytokines that regulate cardiomyocytes and other cell types that are involved in the regeneration and/or healing of injured tissues. Exosomes can also be used as diagnostic markers because their characteristics are similar to those of the tissues they originate from. Utilizing computational 3D models can help change parameters more easily and contribute to the development of more complex systems in the future. Methods for isolating cardiac stem cells have evolved; however, they are still under discussion. Recent studies have suggested that resident macrophages can trigger cell regeneration. As macrophages express chemokine receptors, chemokines are also important in the regulation of macrophages. Exosomes are used for cell-cell communication in macrophages and the surrounding cells. Therefore, macrophages may play a key role in regenerative medicine in the future.

## Data Availability

Not applicable.
